# A Case Report on LUMBAR Syndrome in an Infant With Ulcerated Sacral Hemangioma and Spinal Dysraphism

**DOI:** 10.7759/cureus.80097

**Published:** 2025-03-05

**Authors:** João Soares, Ricardo Craveiro Costa, Rúben Cardoso, Joana Pinto, Sílvia Carvalho, Leonor Castendo Ramos

**Affiliations:** 1 Dermatology, Hospital Pediátrico, Unidade Local de Saúde de Coimbra, Coimbra, PRT; 2 Pediatrics, Hospital Pediátrico, Unidade Local de Saúde de Coimbra, Coimbra, PRT; 3 Neurological Surgery, Hospital Pediátrico, Unidade Local de Saúde de Coimbra, Coimbra, PRT; 4 Medical Imaging, Hospital Pediátrico, Unidade Local de Saúde de Coimbra, Coimbra, PRT

**Keywords:** case report, infantile hemangioma, lumbar syndrome, oral propranolol, spinal dysraphism, tethered spinal cord

## Abstract

Infantile hemangiomas are the most common soft tissue tumors in infancy, most following a benign and predictable course. However, some hemangiomas, particularly those in the lower body, can indicate underlying syndromic anomalies, as seen in lower body hemangiomas and other cutaneous defects, urogenital anomalies, ulceration, myelopathy, bony deformities, anorectal malformations, arterial anomalies, and renal anomalies (LUMBAR) syndrome. This report presents the case of a term infant with a large superficial ulcerated sacral hemangioma and associated spinal dysraphism, including tethered cord, partial sacral agenesis, and intraspinal lipoma. The early multidisciplinary evaluation confirmed the diagnosis of LUMBAR syndrome, and the patient underwent surgical management of cutaneous discontinuities and initiated oral propranolol. Propranolol was effective in resolving the ulcerated component of the hemangioma. This case highlights the importance of recognizing lower body hemangiomas as markers for potential underlying anomalies, emphasizing the role of early diagnosis, comprehensive imaging, and multidisciplinary care in optimizing outcomes for this rare but complex syndrome.

## Introduction

LUMBAR syndrome is a rare but clinically significant constellation of anomalies encompassing lower body hemangiomas and other cutaneous defects, urogenital anomalies, ulceration, myelopathy, bony deformities, anorectal malformations, arterial anomalies, and renal anomalies [1]. Historically, partial features of LUMBAR were described using the acronyms PELVIS [2] and SACRAL [3]; LUMBAR is now considered the most comprehensive term, encompassing all these features [4]. The associated malformations, including spinal dysraphism, tethered cord, lipomas, or sacral agenesis, can be severe. An apparently benign infantile hemangioma may therefore signal more extensive underlying anomalies, highlighting the importance of recognizing LUMBAR as a framework for comprehensive evaluation and care.

Infantile hemangiomas are the most common soft tissue tumors of infancy, observed in approximately 5%-10% of one-year-old infants [5]. Although most follow a benign, self-limited course of proliferation, plateau, and eventual resolution, some are complicated by ulceration or the involvement of internal structures [5]. Ulcerated hemangiomas, particularly in the sacral and gluteal regions, present clinical challenges due to higher risks of infection, bleeding, and delayed healing. Given that these lesions can serve as cutaneous markers of potentially serious syndromic anomalies, such as LUMBAR syndrome, prompt recognition and thorough investigation are crucial for optimizing patient outcomes.

## Case presentation

**Figure 1:**
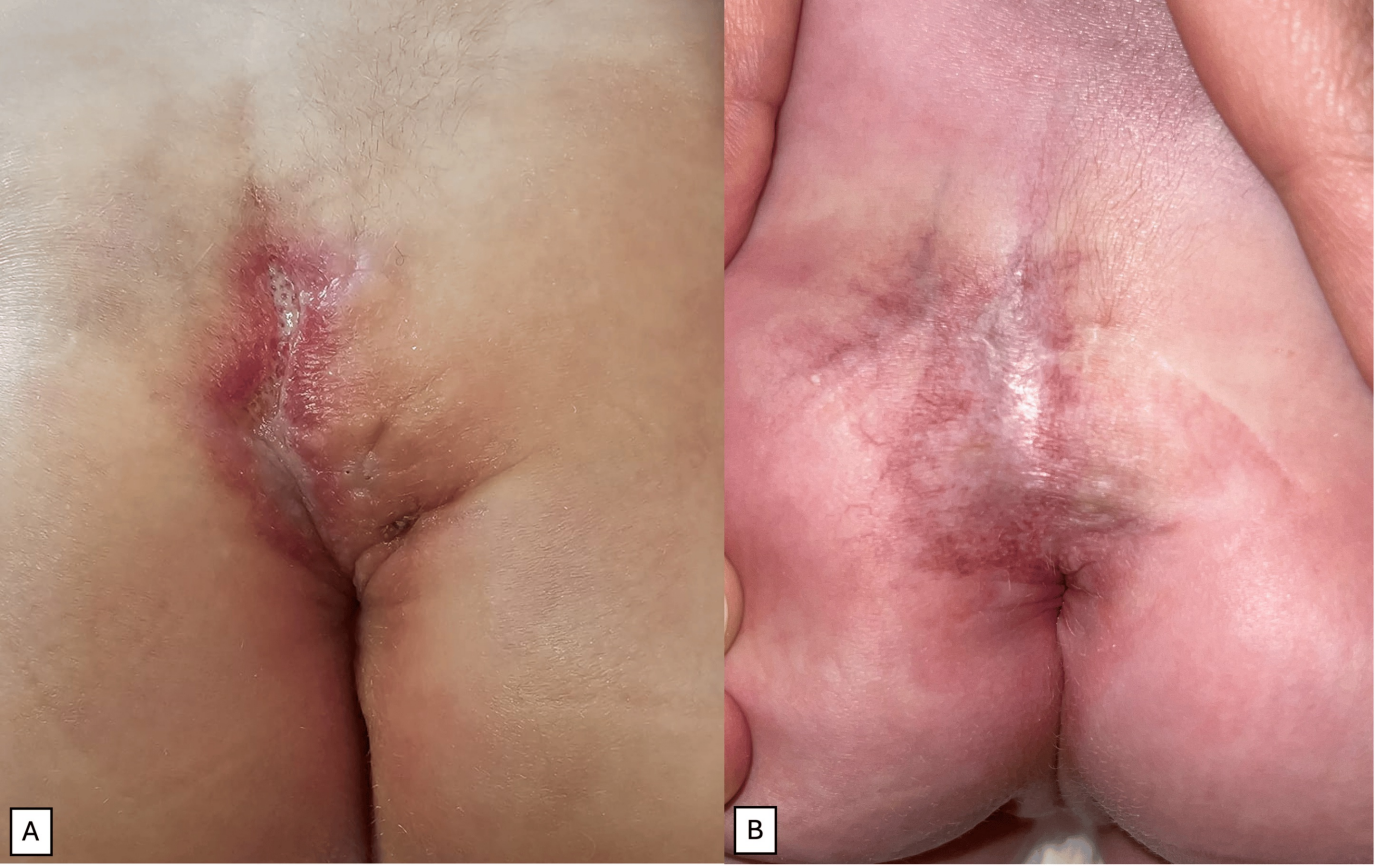
Clinical photographs of the ulcerated hemangioma in the intergluteal cleft. (A) At presentation, showing a large erythematous plaque with central ulceration and a surrounding halo of vasoconstriction. (B) After 10 weeks of propranolol therapy, with persistent erythematous plaque corresponding to an involuting hemangioma and the re-epithelialization of the central ulcer.

**Figure 2:**
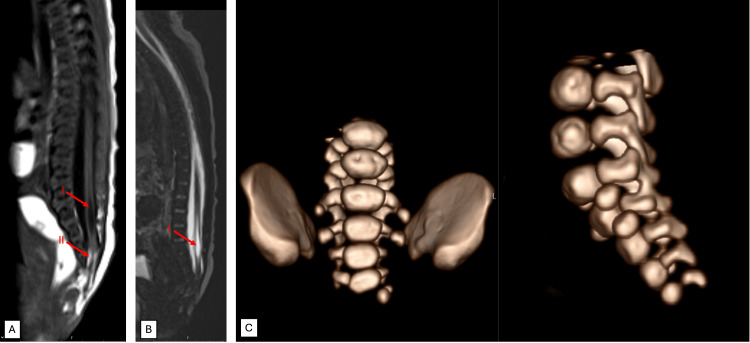
MRI (A and B) and CT (C) findings showing tethered cord, intraspinal lipoma, hydromyelia, and sacral partial agenesis. (A) T1-weighted sagittal magnetic resonance imaging (MRI) showing mild caudal regression with a tethered cord, evidenced by the abnormally low position of the conus medullaris below the L1-L2 level. Additional findings include syringohydromyelia (I) and an intraspinal terminal lipoma contiguous with subcutaneous fat (II). (B) Volumetric 3D T2-weighted sagittal MRI sequence in the sagittal plane demonstrating the tethered cord and syringohydromyelia (I). (C) Volumetric reconstruction of computed tomography (CT) of the lumbosacral spine showing partial agenesis of S5 and agenesis of the coccyx. Note the presence of movement artifacts. These artifacts are inherent challenges in neonatal imaging and may slightly affect the resolution of certain details.

A first-born female term infant was noted at birth to have a large erythematous plaque, approximately 3 cm in its greatest dimension, extending from the sacrococcygeal region into the intergluteal sulcus, with a superficial central ulcer (Figure 1). The lesion had a bright red color, sharply demarcated borders, and a surrounding halo of vasoconstriction, features clinically consistent with infantile hemangioma. No other vascular lesions were identified elsewhere on the body, and there was no family history of similar lesions. Antenatal scans revealed no fetal anomalies, and the mother had received folic acid supplementation prior to conception. The infant had normal bowel and bladder function, normal strength in all limbs, and no anal involvement. Magnetic resonance imaging (MRI) confirmed a closed spinal dysraphism with partial absence of the right S5 vertebra, a tethered spinal cord, and an intraspinal lipoma (Figure 2).

The neurosurgical evaluation noted the need for the closure of two smaller, separate cutaneous discontinuities in the right gluteal region to reduce the risk of infection. This was subsequently addressed surgically, with the closure of the described lesions. Postoperative recovery was unremarkable, and all surgical sites were successfully sealed without complications. Neurological assessments, including evaluation by pediatric neurology, revealed no focal deficits. However, due to the presence of spinal tethering, ongoing monitoring is required, with a surgical intervention planned at the age of two years.

On initial dermatologic evaluation, the suspicion of LUMBAR syndrome was raised, due to the association of the ulcerated hemangioma with the underlying spinal dysraphism and sacral agenesis. Additional investigations, including abdominal and renal ultrasonography, ruled out concurrent genitourinary anomalies.

The patient was started on propranolol therapy at five weeks and five days of age, initiated in a hospital setting, at a dose of 1 mg/kg/day, and titrated to 2 mg/kg/day after one week. Parents reported intermittent sleep disturbance during dose adjustments. Over the following weeks, the ulceration demonstrated progressive re-epithelialization, with complete healing observed during follow-up.

**Figure 3:**
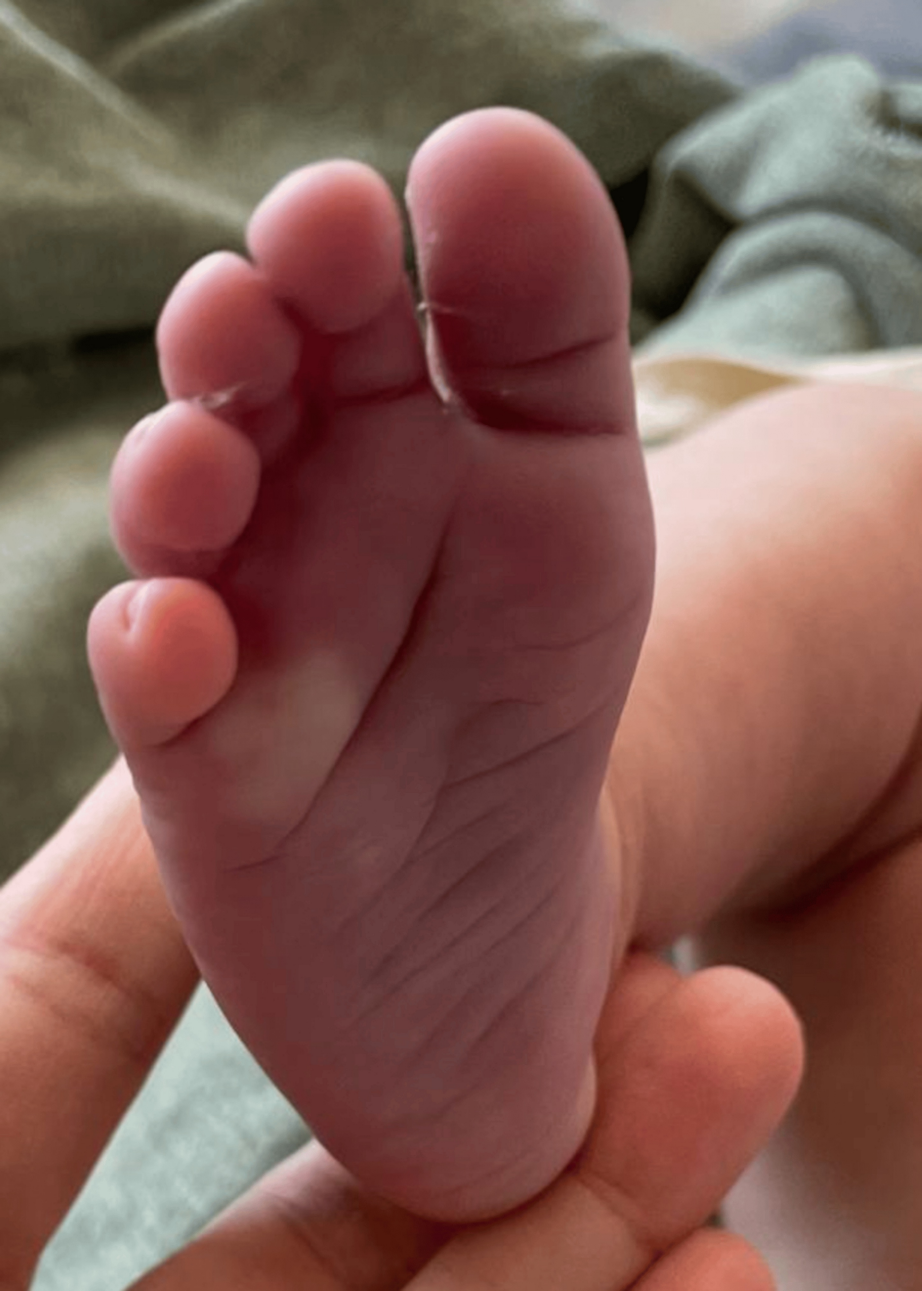
Acrocyanosis of the right foot, characterized by a bluish discoloration and cold extremity.

A Doppler ultrasound study of the lower limbs was performed to assess vascular involvement due to episodes of acrocyanosis in the extremities (Figure 3). Findings revealed normal arterial flow with no evidence of hemodynamically significant stenosis; thus, acrocyanosis was attributed to a propranolol side effect.

## Discussion

This case highlights the importance of recognizing lower body hemangiomas as potential markers for underlying anomalies. Hemangiomas are the cutaneous hallmark of LUMBAR syndrome and are characteristically large and segmental, often displaying a geographic pattern over a defined anatomic region. The sacral region is the most common site for hemangiomas in LUMBAR syndrome, followed by the lumbar area, perineum/genital regions, and the lower extremities [4].

Diagnostic criteria for LUMBAR syndrome require the presence of a segmental infantile hemangioma localized to the lumbosacral, sacrococcygeal, or pelvic cutaneous regions, combined with at least one additional feature, most commonly a spinal cord malformation, as established by the Delphi Consensus on Diagnostic Criteria for LUMBAR syndrome [4]. Associated spinal malformations in LUMBAR syndrome include tethered cord, spinal agenesis resembling spina bifida, and intraspinal lipomas, as observed in this case. It is uncommon for patients to exhibit the full spectrum of anomalies [1]. Diagnostic studies to identify associated anomalies include spinal MRI and abdominal angio-MRI. However, as angio-MRI may not be readily available in many centers, ultrasound may serve as an initial screening alternative, as was done in this case. The findings underscore the necessity of a multidisciplinary approach, in this case involving dermatology, pediatrics, neurology, and neurosurgery.

The pathophysiology of LUMBAR syndrome remains unclear. In contrast, segmental hemangiomas of the upper body are associated with posterior fossa anomalies, hemangioma, arterial anomalies, cardiac anomalies, eye anomalies, and sternal defects (PHACE) syndrome. Some patients display overlapping features of these syndromes, suggesting a shared embryologic pathophysiology [6]. The segmental morphology of cutaneous hemangiomas supports a mosaic phenomenon, potentially linking the hemangiomas to the associated internal anomalies. The aberrant migration of mesenchymal stem cells has been proposed as a key factor in this process [7].

This case also highlights the challenges of managing ulcerated hemangiomas, including the risks of infection, bleeding, and delayed healing. Propranolol, with its vasoconstrictive effects mediated by β_2 _receptor blockade, proved effective in this case. Acrocyanosis, a known side effect of propranolol [8], is also in the phenotypic spectrum of LUMBAR syndrome, since it may also be associated with stenosis/occlusion of major arteries of the lower body [4]. Doppler ultrasound was used to exclude such vascular anomalies in this case, and the symptoms were mild and clinically manageable.

This case is unique due to the simultaneous presence of a large ulcerated hemangioma and spinal dysraphism, in the context of the rarity of LUMBAR syndrome, underscoring the critical importance of early recognition and timely intervention, with a positive treatment response to propranolol.

A limitation of this case is the lack of long-term follow-up data, particularly concerning spinal dysraphism and potential hemangioma recurrence [9]. Future research should focus on studying the natural history of LUMBAR syndrome, optimizing treatment strategies, and investigating potential embryologic mechanisms underlying these syndromes.

## Conclusions

Lower body hemangiomas, particularly when ulcerated or located in the sacral region, are significant diagnostic markers for underlying syndromic conditions such as LUMBAR syndrome and associated anomalies, including spinal dysraphism, tethered cord, and sacral agenesis. Early detection and thorough diagnostic evaluation are essential for optimizing patient outcomes, particularly given the complexity and rarity of these presentations. This case also reinforces the role of propranolol as a first-line treatment in similar scenarios, emphasizing its ability to address complications associated with hemangiomas.

This case highlights the need for further research into the embryologic mechanisms linking cutaneous hemangiomas to internal malformations. Longitudinal studies are also warranted to better understand the natural history of LUMBAR syndrome and its optimal management.

## References

[1] Iacobas I, Burrows PE, Frieden IJ (2010). LUMBAR: association between cutaneous infantile hemangiomas of the lower body and regional congenital anomalies. J Pediatr.

[2] Girard C, Bigorre M, Guillot B, Bessis D (2006). PELVIS syndrome. Arch Dermatol.

[3] Stockman A, Boralevi F, Taïeb A, Léauté-Labrèze C (2007). SACRAL syndrome: spinal dysraphism, anogenital, cutaneous, renal and urologic anomalies, associated with an angioma of lumbosacral localization. Dermatology.

[4] Metry D, Copp HL, Rialon KL (2024). Delphi Consensus on Diagnostic Criteria for LUMBAR syndrome. J Pediatr.

[5] Drolet BA, Esterly NB, Frieden IJ (1999). Hemangiomas in children. N Engl J Med.

[6] Davenport R, Su JC, Nathalie J, Richmond CM, Yang Tan T, Robertson SJ (2022). Clinical overlap of PHACE and LUMBAR syndromes. Pediatr Dermatol.

[7] Stefanko NS, Davies OM, Beato MJ (2020). Hamartomas and midline anomalies in association with infantile hemangiomas, PHACE, and LUMBAR syndromes. Pediatr Dermatol.

[8] Marqueling AL, Oza V, Frieden IJ, Puttgen KB (2013). Propranolol and infantile hemangiomas four years later: a systematic review. Pediatr Dermatol.

[9] Frongia G, Byeon JO, Mehrabi A, Günther P (2021). Recurrence rate of infantile hemangioma after oral propranolol therapy. Eur J Pediatr.

